# Kai-Xin-San ameliorates mild cognitive impairment in SAMP8 mice by inhibiting neuroinflammation and pyroptosis via NLRP3/Caspase-1 pathway modulation

**DOI:** 10.3389/fphar.2025.1528011

**Published:** 2025-03-13

**Authors:** Shu Liu, Xiaochen Song, Yuefeng Sun, Ailin Sun, Yang Li, Yuyu Li, Jing Chen

**Affiliations:** ^1^ College of Basic Medical and Sciences, Heilongjiang University of Chinese Medicine, Harbin, Heilongjiang, China; ^2^ The Faculty of Medicine, Qilu Institute of Technology, Jinan, Shandong, China

**Keywords:** Kai-Xin-San, TCM, mild cognitive impairment, anti-inflammation function, NLRP3 inflammasome, pyroptosis

## Abstract

Mild Cognitive Impairment (MCI) represents a critical stage between normal aging and dementia, with limited effective interventions currently available. This study investigated the effects of Kai-Xin-San (KXS), a traditional Chinese herbal formula, on cognitive function, neuroinflammation, and pyroptosis in a senescence-accelerated prone 8 (SAMP8) mouse model of MCI. SAMP8 mice were treated with KXS for 8 weeks, followed by behavioral tests, biochemical analyses, and histological examinations. KXS significantly improved spatial memory, working memory, and executive function in SAMP8 mice. Furthermore, KXS treatment reduced β-amyloid (Aβ) deposition, attenuated neuroinflammation by decreasing pro-inflammatory cytokine levels (IL-1β, IL-18, IL-6, TNF-α), and inhibited microglia activation in the hippocampus. Notably, KXS suppressed pyroptosis by modulating the NLRP3/Caspase-1 signaling pathway, as evidenced by reduced expression of NLRP3, ASC, Caspase-1, and GSDMD. These effects were abolished by treatment with the NLRP3 inflammasome agonist Nigericin, suggesting that NLRP3 inhibition is a key mechanism of KXS action. Our findings reveal a novel mechanism by which KXS exerts neuroprotective effects in MCI, simultaneously targeting Aβ accumulation, neuroinflammation, and pyroptosis. This multi-target approach of KXS highlights its potential as a therapeutic strategy for MCI and warrants further investigation in clinical settings.

## Highlights

• Kai-Xin-San (KXS) can prevent and treat mild cognitive impairment (MCI).
• KXS improved spatial and working memory and reduced brain injury in SAMP8 mice.
• KXS showed therapeutic potential for MCI in SAMP8 mice by reducing inflammation.
• KXS treated MCI in SAMP8 mice by inhibiting the NLRP3/Caspase-1 pyroptosis pathway.

## 1 Introduction

With the accelerating aging population worldwide, diseases related to cognitive impairments such as dementia have become a growing concern for global public health (Jongsiriyanyong and Limpawattana, 2018). Alzheimer’s disease (AD), the predominant type of dementia, impacts countless people worldwide (Selkoe, 2002; Avila and Perry, 2021). It leads to deterioration of cognitive function, reduced quality of life, and loss of independence in advanced stages, posing a serious threat to the health and wellbeing of the elderly population.

Mild cognitive impairment (MCI), characterized by subtle declines in memory and thinking abilities without significant impact on daily activities, represents a critical juncture between normal aging and dementia (Chandra et al., 2019). The importance of focusing on MCI cannot be overstated, as it offers a potential window for intervention before the onset of irreversible AD, it will be irreversible, for which no curative treatments currently exist. This aligns with the traditional Chinese medicine principle of “treating the future disease,” emphasizing early intervention and prevention.

Kai-Xin-San (KXS), a traditional Chinese medicine prescription recorded in the “Bei Ji Qian Jin Yao Fang” by Sun Simiao, a Tang Dynasty physician, has garnered attention for its potential to treat forgetfulness. It is composed of four traditional Chinese botanical drugs, namely, Wolfiporia cocos (F.A. Wolf) Ryvarden and Gilb [Polyporaceae], Panax ginseng C.A.Mey [Araliaceae], Polygala tenuifolia Willd [Polygalaceae], and Acorus tatarinowii Schott [Acoraceae], which is a representative formula for benefiting the intellect and tranquilizing the mind. Recent research has revealed its multifaceted pharmacological effects, including improvements in cognitive function and memory, along with antioxidant and anti-inflammatory capabilities, and enhancement of neurotrophic factors (Luo et al., 2020; Wang et al., 2020; Jiao et al., 2022; Su et al., 2023). The results of the mass spectrometry analysis demonstrated the presence of a total of 77 phyto ingredients, including 26 saponins, 13 triterpenoids, 20 oligosaccharides, 5 ketone compounds, and 13 other components, in the four botanical drugs of KXS. Among these bioactive natural components, 25 were derived from Polygala tenuifolia, primarily comprises oligosaccharides and ketone compounds; 28 were derived from Panax ginseng, predominantly saponins; 17 were derived from Poria cocos, primarily triterpenoid acids; and 5 were derived from Acorus tatarinowii, majorly β-as aryl ether, and other constituents (Li H. et al., 2022). In the analysis, LIN R. et al. employed the UPLC-Q-Orbitrap-MS technique in conjunction with a local database to determine different phytochemicals from KXS. A total of 211 compounds were identified from KXS, including 60 Panax ginseng, 40 Poria cocos, and 111 Poria cocos. Subsequently, 105 volatile compounds were recognized by GC-MS analysis, predominantly originating from the rhizome of Acorus tatarinowii (Lin et al., 2021). Furthermore, ginsenoside Rb1, ginsenoside Re, ginsenoside Rg1, Sibiricose A5, Sibiricose A6, 3,6′-Disinapoylsucrose, polygalaxanthone III, α-asarone, β-asarone, and Poria acid were identified in the serum of rats administered KXS groups (Gao and Lv, 2021). The aforementioned studies have collectively revealed that the components present in KXS, including polygala oligosaccharide esters, ginsenosides, and Acorus volatile oil, could serve as quality markers for KXS.

The pathogenesis of MCI and its progression to AD involve complex mechanisms, with β-amyloid (Aβ) deposition playing a central role. It is a significant contributor to the loss of neurons and the deterioration of cognitive abilities (Hardy and Selkoe, 2002). Excessive Aβ accumulation not only forms neurotoxic senile plaques but also triggers chronic neuroinflammation, creating a detrimental feedback loop that accelerates cognitive decline (Zhang et al., 2019; Li Z. et al., 2021). Furthermore, Aβ can activate NOD-like receptor protein 3 (NLRP3) inflammasome and drive pyroptosis, a recently recognized form of inflammatory cell death that exacerbates neuroinflammation and potentially hastens MCI progression (Yu et al., 2021).

Preliminary studies suggest that KXS may exert its cognitive-enhancing effects in senescence-accelerated prone 8 (SAMP8) mice by modulating Aβ production and catabolism, thereby reducing Aβ deposition and ameliorating pathological changes in brain tissue. Additionally, metabolomic and pharmacological investigations indicate that KXS can improve neuroinflammation and cognitive function by regulating inflammatory mediators (Tangalos and Petersen, 2018; Sun et al., 2021; Wang et al., 2021). However, the specific signaling pathway through which KXS inhibits neuroinflammation and pyroptosis in the context of MCI remains unexplored.

This study aimed to elucidate the capacity of KXS in reducing neuroinflammation and pyroptosis in MCI and to uncover its underlying mechanism. By focusing on the neuroprotective mechanisms underlying the cognitive benefits of KXS, our goal is to offer a thorough insight into its healing possibilities for MCI, bridging traditional wisdom with modern molecular insights.

## 2 Materials and methods

### 2.1 Materials

KXS were procured from Hebei Quantai Pharmaceutical Co., Ltd., (Hebei, China), adhering to the standards of the Pharmacopoeia of the People’s Republic of China (2020 Edition), Part I and Part IV. Donepezil hydrochloride was obtained from Eisai (China) Pharmaceutical Co. Ltd., Fluoro-Jade C (FJC) kit and Congo red staining reagents were obtained from Beijing Solarbio Science and Technology Co., Ltd., (Beijing, China). Vectastain Elite ABC-HRP Immunohistochemistry (IHC) kit was acquired from VECTOR (California, United States). Terminal labeling (TUNEL) apoptosis kit was obtained from Roche (Basel, Switzerland). Hematoxylin, eosin reagent, and the BCA protein concentration determination kit were obtained from Beyotime Biotech. Inc., (Shanghai, China). Sources of ELISA kits for cytokines including IL-18, IL-6, IL-1β, and TNF-α were Elabscience Biotechnology Co., Ltd., and ABclonal Technology Co., Ltd., (both Wuhan, China). Primary antibodies: anti-ASC (cat. no. A16672), anti-Caspase-1 (cat. no. A0964), anti-IL-1β (cat. no. A19635), anti-IL-18 (cat. no. A20473), anti-β-actin (cat. no. AC026), and HPR goat anti-rabbit IgG (cat. no. AS014) from ABclonal Technology Co., Ltd. (Wuhan, China); Anti-Iba-1 (cat. no. DF6442), and anti-Gasdermin D (GSDMD) (cat. no. AF4012) from Affinity Biosciences Co., Ltd. (Jiangsu, China); Goat anti-rabbit IgG (cat. no. Ap132p) from Millipore (Massachusetts, United States); Anti-NLRP3 (cat. no. 381207) from Zen-Bioscience Co., Ltd., (Chengdu, China); Anti-Aβ1-42 (cat. no. BB02112732) from Beijing Biosynthesis Biotechnology Co., Ltd., (Beijing, China).

### 2.2 Preparation of KXS

KXS was prepared using Panax ginseng, Poria cocos, Polygala tenuifolia, and Acorus tatarinowii to crush into fine powder in the ratio of 2:1:1:1, ensuring that not less than 95% of the weight of the medicinal powder passed through a No. 6 sieve (100 mesh). The powders were mixed to create the final powder dosage form.

### 2.3 Animals and drug administration

Male SAMP8 and SAMR1 mice, aged 7 months and sharing the same genetic lineage (weight: 20 ± 2 g), were sourced from the Department of Laboratory Animal Science, Faculty of Medicine, Peking University [SCXK (Beijing) 2016–0,010]. Mice were housed individually in SPF-grade conditions at the Center for Evaluation of Pharmaceutical Safety of the Heilongjiang University of Chinese Medicine, with controlled room temperature (20.0 ∼24.0)°C, humidity of (40.0 ∼ 60.0)%, light/dark cycle of 12 h/12 h. All procedures conducted in the study adhered to the Declaration of Helsinki principles for animal research and complied with the Requirements for Ethical Experiments on Animals and the Guidelines for Experimental Animals and Code of Practice for the Use of Experimental Animals of the Heilongjiang University of Chinese Medicine.

All mice were acclimatized for 7 days and then trained in the Morris water maze. SAMP8 mice with an escape latency of >80 s were selected as MCI experimental animals (Hou et al., 2020) and randomly assigned to the following groups: KXS group (KXS was prepared in distilled water for oral administration at a daily dose of 0.58 g/kg), donepezil group (donepezil hydrochloride was prepared in distilled water and given orally at a daily dose of 0.65 mg/kg), KXS + Nigericin group (Nigericin was prepared in 0.9% saline solution for intraperitoneal injection at a dosage of 1.4 mg/kg, administered every 3 days, and at the same time, KXS was administered by gavage once a day, with an interval of half an hour between the two administrations), and SAMP8 group. The SAMR1 mice were kept in the control group. Mice in the SAMP8 and control groups received an equivalent volume of distilled water orally daily, and each group of mice was gavaged continuously for 28 days.

### 2.4 Behavioral tests

#### 2.4.1 Morris water maze (MWM) test

MCI model animals were screened by the MWM test 7 days before the start of formal experiments. The apparatus consisted of a circular pool measuring 120 cm in diameter and 45 cm in depth, with black-painted interior walls. A video camera above the pool recorded swimming trajectories, which were analyzed by computer software to calculate escape latency, swimming distance, and speed. Each mouse underwent four separate trials daily, with a 10-min interval between each trial and a 90-s limit per trial. Following each trial, they were permitted to remain on the platform for 30 s.

The localization navigation experiment was conducted for 5 consecutive days, starting from the 21st day of administration. Randomly selected quadrants were used as starting points, where mice were released into the water to swim and locate the hidden platform. When a mouse located and climbed onto the platform within the 120-s timeframe, it was permitted to remain there for 10 s, and the latency was noted. In case of not finding the safe platform, the mouse was artificially guided to the platform for 10 s, so that it could memorize the platform’s position, and the recording time was 120 s. The space exploration experiment was conducted the next day after the localization navigation experiment, and the duration of the experiment was 1 day. Then the safety platform was taken out of the pool, the mouse was put into the water. The frequency with which the mouse crossed the former platform’s location within 120 s was documented, serving as a measure of its spatial memory capacity.

#### 2.4.2 Nest-building test

Prior to the experiment, old bedding was removed from the cages. Each cage was then prepared with 20 g of fresh shavings, topped with 16 sheets of thin white tissue paper (4.5 cm × 4.5 cm). Mice were allowed 12 h for nest building, after which their nests were scored. The scoring criteria for the nest-building test are shown in Table 1. This test provides a measure of the mice’s cognitive function and motivation, with higher scores indicating better performance.

**TABLE 1:** Nesting-building score scale (Deacon, 2006).

Score	Scoring standard
1	There were no tear marks on the tissue paper
2	A small portion of tissue paper was torn
3	A large portion of tissue paper was torn, but no recognizable nests were formed
4	Most of the tissue paper was torn and had been built into flatter nests
5	The tissue paper was completely nested

#### 2.4.3 Novel object recognition (NOR) test

The NOR test took place in a square arena (40 cm × 40 cm × 40 cm) over two consecutive days. A video camera positioned above the box recorded the mice’s movements. On the first day, mice were permitted to adjust to the surroundings for 10 min before being returned to their home cages. On the second day, they underwent familiarization and testing phases. During familiarization, two identical items (A1 and A2) were positioned in the arena for a 5-min exploration period, during which the time spent interacting with each item was documented. In the test phase, 1 hour later, A2 was replaced with a new object (B) in the same spot. Mice were again allowed 5 min of exploration, with their interaction times recorded. The recognition index, determined by dividing the exploration time of the novel object B by the total exploration time for objects A1 and B, was utilized to evaluate object recognition memory.

### 2.5 Tissue collection

Following behavioral tests, mice were anesthetized with 1% sodium pentobarbital administered intraperitoneally, with dosage adjusted to body weight. Blood was collected via retro-orbital bleeding, centrifuged to separate serum, and then stored at −80°C for ELISA analysis. The hippocampi were extracted and sectioned into three parts: one portion was immersed in 4% paraformaldehyde at room temperature for subsequent paraffin embedding, another part was preserved in glutaraldehyde fixative at 4°C for transmission electron microscopy, and the remaining tissue was flash-frozen and stored at −80°C for Western blotting analysis.

### 2.6 Histological staining

Paraffin-embedded hippocampal tissues were sectioned at 4-μm-thickness. After deparaffinization and hydration, sections underwent three separate staining procedures: FJC staining, Congo red staining, and hematoxylin-eosin (H&E) staining. All staining procedures followed the respective kit instructions. After staining, sections were dehydrated, cleared, and mounted with neutral gum. Stained sections were stained and examined microscopically (BX53, OLYMPUS, Japan) for analysis.

### 2.7 TUNEL staining

Paraffin-embedded hippocampal sections were routinely deparaffinated and rehydrated. The sections were then treated successively with a proteinase K working solution and membrane stripping solution. The TUNEL method was followed as per the kit’s protocol to perform TUNEL staining. Subsequently, nuclei were stained with DAPI. The stained sections were mounted using a fluorescence anti-quenching agent and examined under the microscope.

### 2.8 IHC

Hippocampal sections underwent deparaffinization, rehydration, and antigen retrieval, followed by blocking with a BSA solution and incubation with primary antibodies. Immunoreactivity was visualized using a DAB substrate solution followed by hematoxylin counterstaining. The stained sections were dehydrated and clarified, mounted with neutral gum, and analyzed microscopically.

### 2.9 Transmission electron microscopy (TEM)

The fixed tissue samples were dehydrated, infiltrated in a mixture of embedding agent and acetone, and embedded using an epoxy resin embedding agent to form a hard resin-embedded block. The resin-embedded blocks were stained after being cut into 7-μm-thick sections and finally observed under a TEM.

### 2.10 ELISA

Before the start of the test, all reagents were moved to room temperature to equilibrate for 30 min before use. According to the number of samples in the experiment, the required slats were taken from the aluminum foil pouch, and the remaining slats were stored at 4°C after sealing them well. IL-1β, IL-18, IL-6, and TNF-α concentrations in Response to question 1 of reviewer 3 the mouse serum were determined following the ELISA kit protocol.

### 2.11 Western blotting

The hippocampi were homogenized in lysis buffer, centrifuged at 12,000 rpm for 5 min, and protein concentrations were determined with a BCA kit. Samples were prepared with loading buffer and denatured at 100°C for 15 min. Proteins were resolved by SDS-PAGE, transferred to membranes, and blocked with 5% non-fat milk for 1 h at room temperature. They were then incubated with the primary antibody at 4°C overnight, followed by incubation with the secondary antibody for 2 h at room temperature. Bands were detected with a chemiluminescent substrate and captured using an imaging system (Beijing SAIZ Technology Co., Ltd., Beijing, China).

### 2.12 Statistical analysis

Data analysis was conducted using SPSS 26.0 and GraphPad Prism 10.1.2. Results are presented as mean ± SD. Statistical evaluations were carried out with one-way ANOVA or suitable non-parametric tests. Significance was set at P < 0.05.

## 3 Results

### 3.1 KXS enhances cognitive function in SAMP8 mice

An animal model of MCI was established by using SAMP8 mice, which are characterized by a spontaneous rapid aging phenotype and short lifespan. SAMP8 mice of 7-month-old were selected to study the inflammatory response as well as pyroptosis of the hippocampus followed by KXS treatment.

**FIGURE 1:**
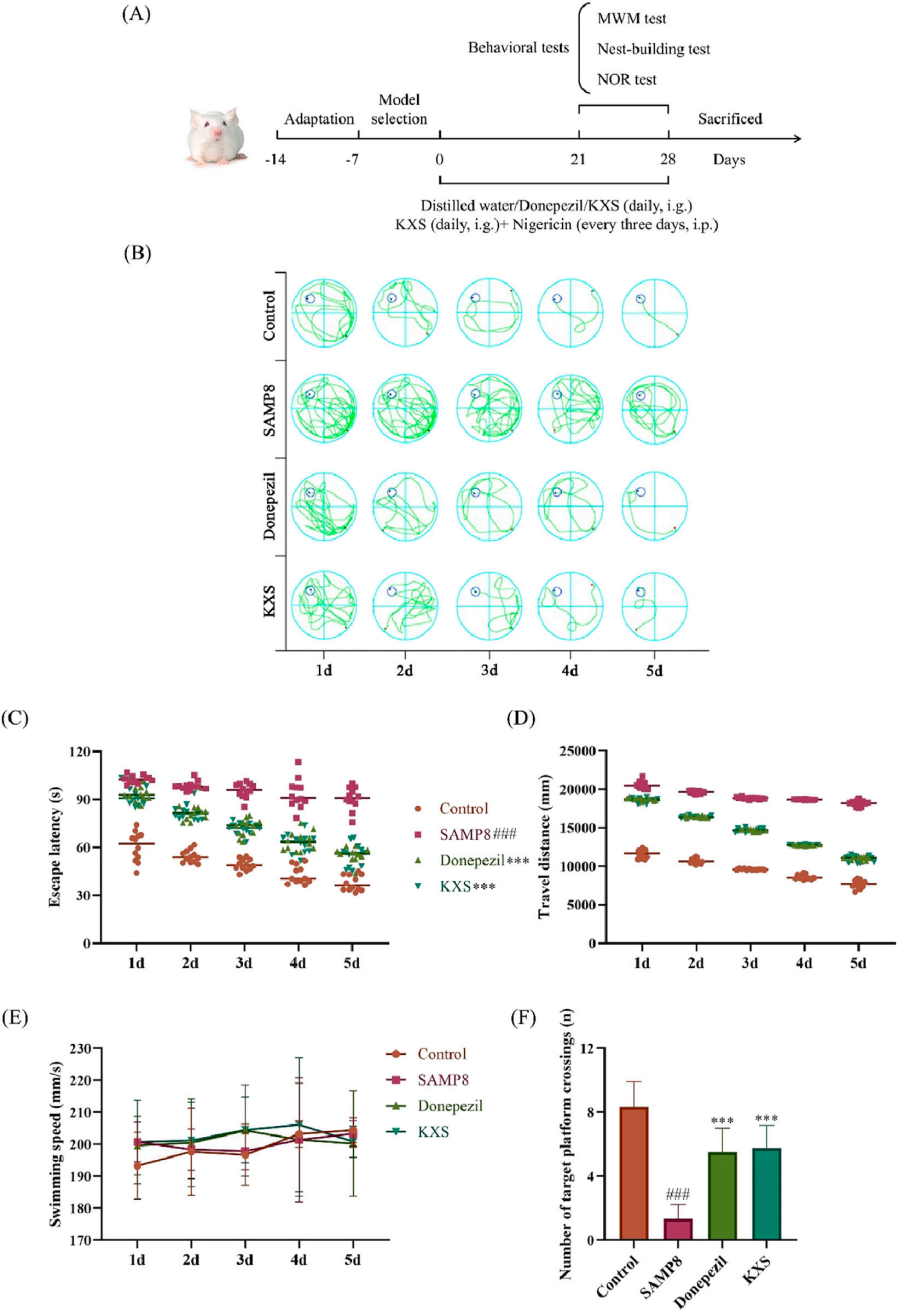
KXS improved learning and spatial memory abilities in SAMP8 mice (n = 12). **(A)** Experimental timeline. **(B)** Representative swimming trajectories during the localization navigation test. **(C)** Escape latency in the localization navigation test. **(D)** Travel distance in the localization navigation test. **(E)** Swimming speed in the localization navigation test. **(F)** Platform crossing in the spatial exploration test. Results are expressed as mean ± SD. ^###^ *P* < 0.001 vs the control group; ***P < 0.001 vs. the SAMP8 group.

First, we conducted a thorough assessment of KXS’s impact on cognitive performance in SAMP8 mice through three behavioral assays: the MWM test, the nest-building test, and the NOR test, as outlined in Figure 1A. The MWM was utilized to evaluate the learning and spatial memory capabilities of the SAMP8 mice. As shown in the localization navigation experiment (Figures 1B–D), The SAMP8 mice exhibited notably extended escape latency and swimming distance compared to the SAMR1 control group, suggesting substantial deficits in spatial learning and memory among SAMP8 mice. Notably, KXS treatment markedly improved these deficits. After treatment with KXS, the escape latency and swimming distance in SAMP8 mice were considerably shortened. With no significant variation in swimming speed among the groups, this suggests that the experimental treatment did not lead to exhaustion or affect the motor function of the mice (Figure 1E). In the spatial exploration experiment, the frequency of platform crossings by SAMP8 mice was markedly lower than that of the control group. KXS treatment notably enhanced the number of platform crossings in SAMP8 mice, indicating improved spatial memory retention (Figure 1F).

**FIGURE 2:**
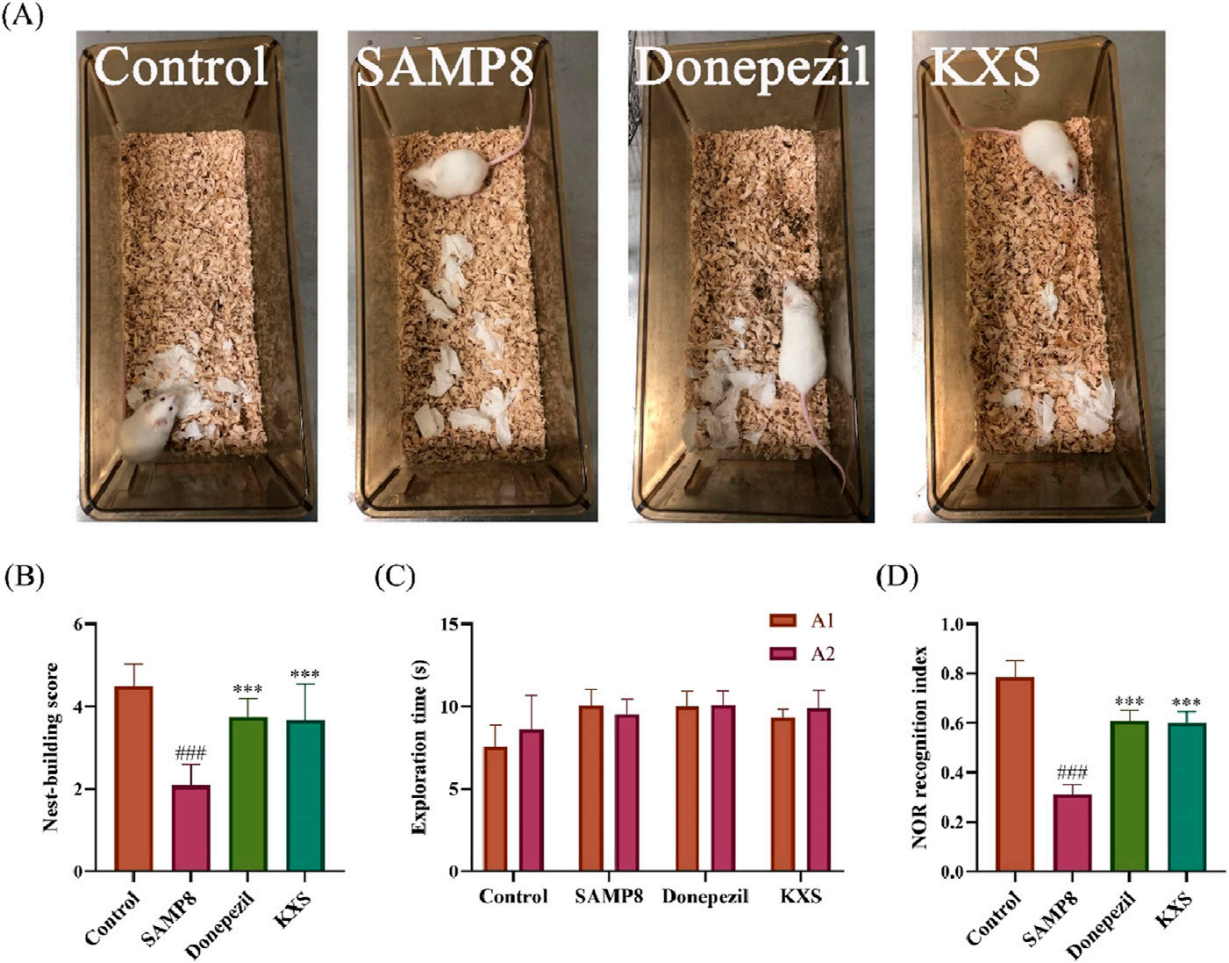
KXS ameliorated brain damage and enhanced working memory in SAMP8 mice (n = 12). **(A)** Representative nest-building test pictures. **(B)** The nest-building test scores. **(C)** Object exploration time during the familiarization period of the NOR test. **(D)** Recognition index during the test period of the NOR test. Results are expressed as mean ± SD. ^###^ *P* < 0.001 vs. the control group; ***P < 0.001 vs. the SAMP8 group.

Nest-building ability is an indicator of brain damage in mice (Deacon, 2012). As shown in the nest-building test (Figure 2A), SAMP8 mice exhibited significantly lower nest-building scores compared to SAMR1 controls. Remarkably, KXS treatment restored nest-building performance in SAMP8 mice to >90% levels of the control group, which is similar to that of the donepezil treatment (Figure 2B).

The NOR test revealed deficits in object discrimination memory in SAMP8 mice. During the familiarization phase, all groups showed similar exploration times for the identical objects A1 and A2, indicating no bias (Figure 2C). In the testing phase, mice in the SAMP8 group exhibited a markedly reduced recognition index for novel objects when compared to the control group. The recognition index of SAMP8 mice to novel objects after treatment with KXS and donepezil was significantly improved (Figure 2D), suggesting that KXS enhanced the discriminative memory of SAMP8 mice to novel objects.

### 3.2 KXS improves hippocampal neuronal degeneration in SAMP8 mice

**FIGURE 3:**
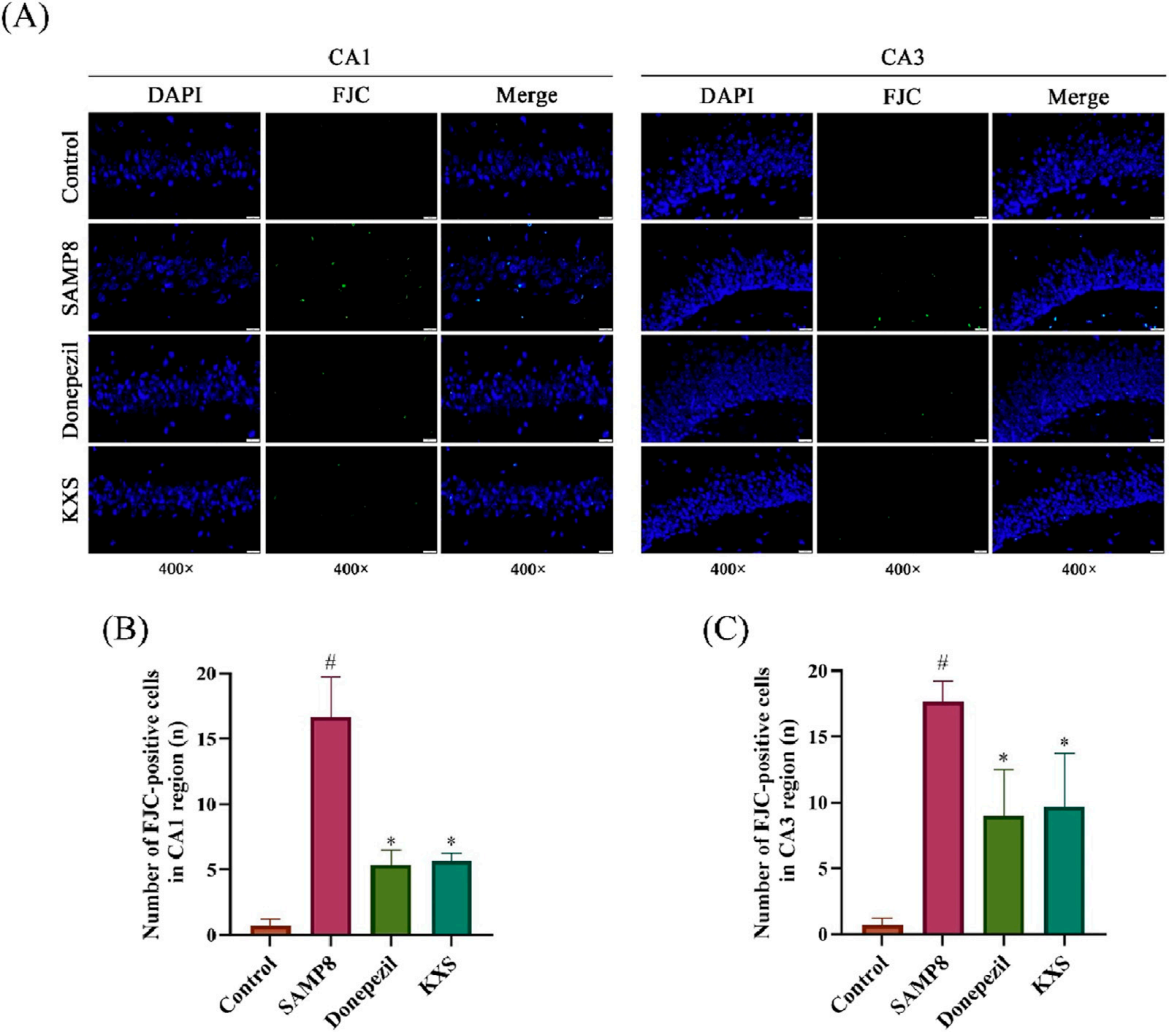
KXS reduced degeneration of hippocampal neurons in SAMP8 mice (n = 3). **(A)** Typical micrographs of FJC staining in the hippocampal CA1 and CA3 regions. Magnification, ×400. Scale bar, 20 μm. **(B)** Counting of FJC-positive cells in the CA1 region. **(C)** Counting of FJC-positive cells in the CA3 region. Results are expressed as mean ± SD. ^#^ *P* < 0.05 vs. the control group; *P < 0.05 vs. the SAMP8 group.

To determine how KXS provides cognitive benefits through neuroprotection, we assessed several hippocampal neuropathological markers in SAMP8 mice. To assess the extent of neuronal damage, we employed FJC staining, a highly sensitive and specific method for detecting degenerating neurons (Ikenari et al., 2020). FJC staining revealed a significant increase of degenerated nerve cells (green fluorescence) in the hippocampal CA1 and CA3 areas of SAMP8 mice. Treatment with KXS significantly decreased the count of FJC-positive cells in these regions, and donepezil groups showed similar reduction levels (Figures 3A–C).

### 3.3 KXS reduces hippocampal amyloid deposition in SAMP8 mice

**FIGURE 4:**
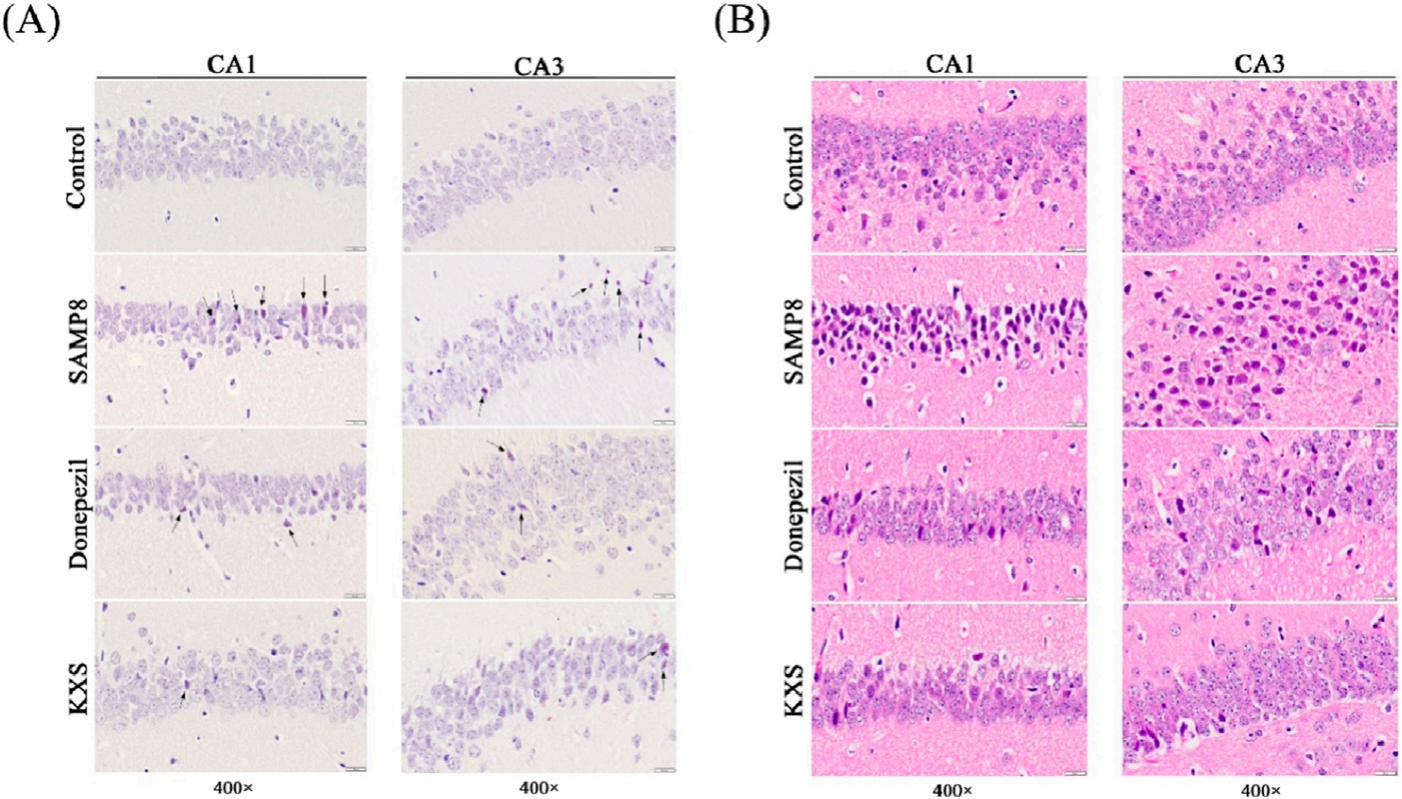
KXS reduced hippocampal amyloid deposition and neuropathologic damage in SAMP8 mice (n = 3). **(A)** Typical micrographs of Congo red staining in the hippocampal CA1 and CA3 regions. **(B)** Typical micrographs of H&E staining in the hippocampal CA1 and CA3 regions. Magnification, ×400. Scale bar, 20 μm.

To visualize amyloid deposits, we used Congo red staining, which specifically binds to amyloid fibrils (Sarkar et al., 2020). Congo red staining showed extensive amyloid deposits with scattered distribution (reddish or date-red color) in the hippocampal CA1 and CA3 regions of SAMP8 mice, and nothing was presented in the control group (Figure 4A). After treatment with KXS and donepezil, an evident reduction of amyloid deposits was observed. Compared to the donepezil group, KXS seems to have a stronger effect (Figure 4A).

### 3.4 KXS attenuates hippocampal neuropathologic damage in SAMP8 mice

To evaluate overall tissue architecture and cellular morphology, we performed H&E staining, a classic histological technique. As shown in Figure 4B H&E staining showed that the cytoarchitecture of the CA1 and CA3 regions of the hippocampus of SAMP8 mice was blurred compared with that of the control group. Neuronal count was diminished, with cells haphazardly arranged and dispersed, alongside cytoplasmic expansion or distortion; various types of neuroglia were irregular in morphology and appeared to be markedly proliferated. KXS or donepezil treatment significantly ameliorated these changes, restoring neuronal density, organization, and morphology to near-normal levels. The number of neuronal cells increased significantly, evenly distributed, and the pathological changes such as pyknosis and displacement of the nucleus were greatly improved; the morphology of neuroglia tended to be normalized.

### 3.5 KXS reduces cell death in the hippocampus of SAMP8 mice

**FIGURE 5:**
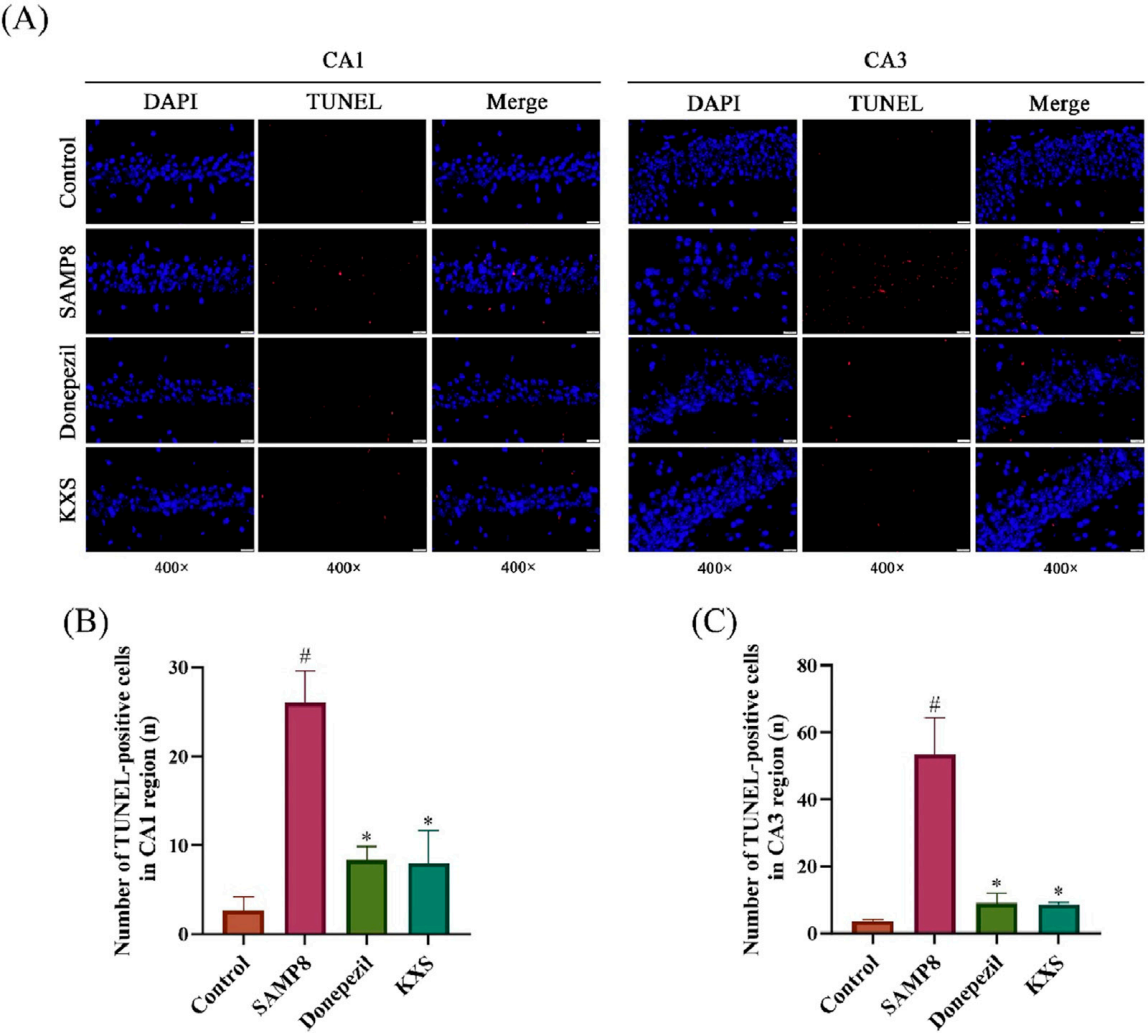
KXS decreased hippocampal neuronal apoptosis in SAMP8 mice (n = 3). **(A)** Typical micrographs of TUNEL staining in the hippocampal CA1 and CA3 regions. Magnification, ×400. Scale bar, 20 μm. **(B)** Counting of TUNEL-positive cells in the CA1 region. **(C)** Counting of TUNEL-positive cells in the CA3 region. Results are expressed as mean ± SD. ^#^ *P* < 0.05 vs. the control group; *P < 0.05 vs. the SAMP8 group.

To investigate the extent of cell death, including both apoptosis and pyroptosis, we utilized TUNEL staining, which detects DNA fragmentation characteristic of dying cells. As shown in Figure 5A, the TUNEL staining indicated a substantial rise in the number of positive cells, as evidenced by red fluorescence, in the hippocampal CA1 and CA3 regions of SAMP8 mice when compared to the SAMR1 control group. Treatment with KXS significantly decreased TUNEL-positive cell counts in these hippocampal areas, with reductions comparable to those observed in the donepezil group (Figures 5A–C). These results suggest that KXS exerts neuroprotective effects by decreasing the pyroptosis of hippocampal cells and preventing hippocampal cell death.

### 3.6 KXS reduces the activation of hippocampal microglia in SAMP8 mice

**FIGURE 6:**
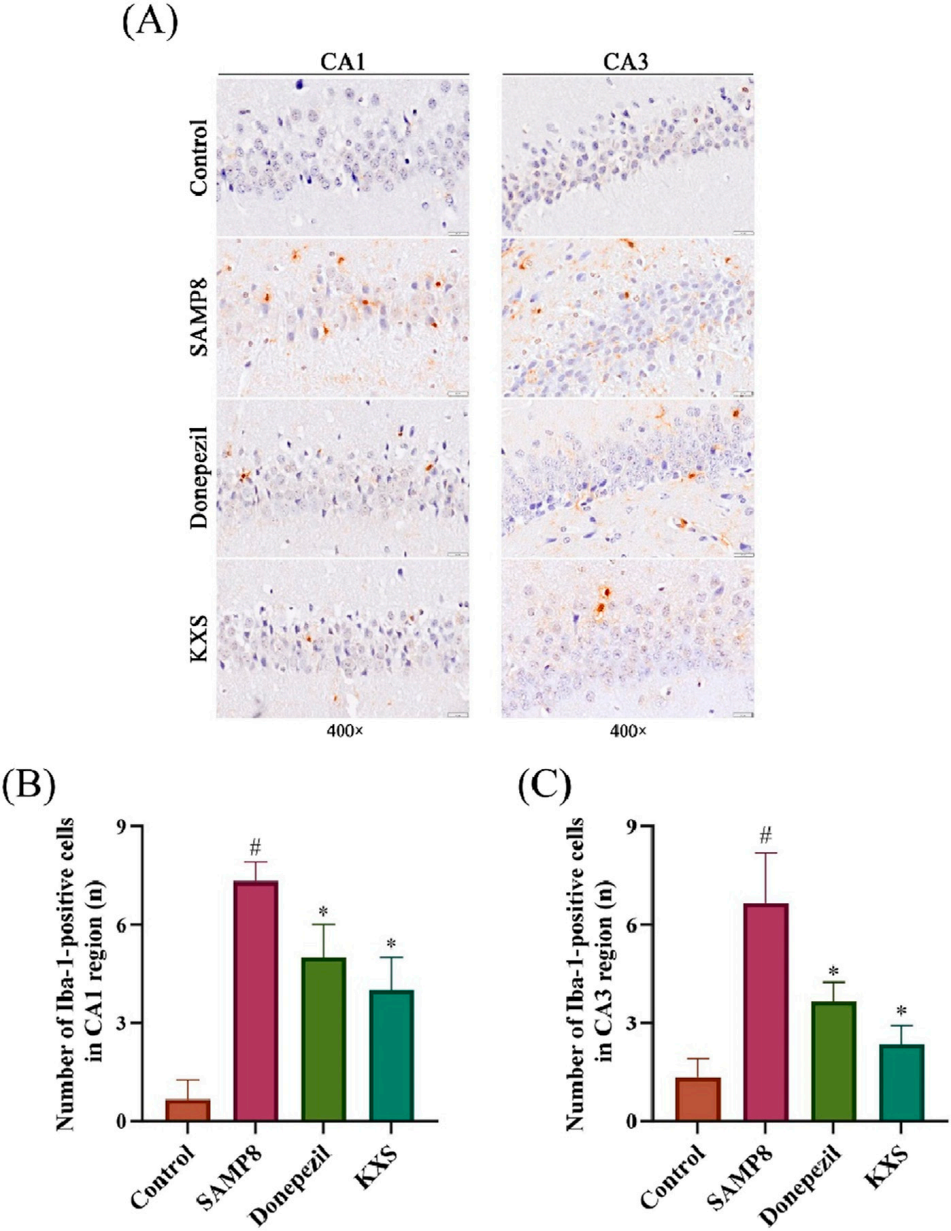
KXS reduced the activationof hippocampal microglia in SAMP8 mice (n = 3). **(A)** Typical micrographs of Iba-1 immunohistochemical staining in the hippocampal CA1 and CA3 regions. Magnification, ×400. Scale bar, 20 μm. **(B)** Counting of Iba-1-positive cells in the CA1 region. **(C)** Counting of Iba-1-positive cells in the CA3 region. Results are expressed as mean ± SD. ^#^ *P* < 0.05 vs. the control group; *P < 0.05 vs. the SAMP8 group.

Microglial activation, a hallmark of neuroinflammation, significantly contributes to cognitive deterioration associated with aging. To assess microglial activation, we performed immunohistochemical staining for Iba-1, a microglial calcium-binding protein unique to the central nervous system (CNS) microglia whose expression increases when microglia are activated (Xiao et al., 2022). As depicted in Figure 6A, Iba-1-positive cell counts, characterized by rust or brownish-yellow coloration, were notably elevated in the hippocampal CA1 and CA3 regions of SAMP8 mice relative to the control group. Treatment with KXS or donepezil significantly lowered the quantity of Iba-1-positive cells in the hippocampal CA1 and CA3 areas, signifying an anti-neuroinflammatory effect (Figures 6A–C).

### 3.7 KXS reduces pyroptosis in SAMP8 mice hippocampal cells

**FIGURE 7:**
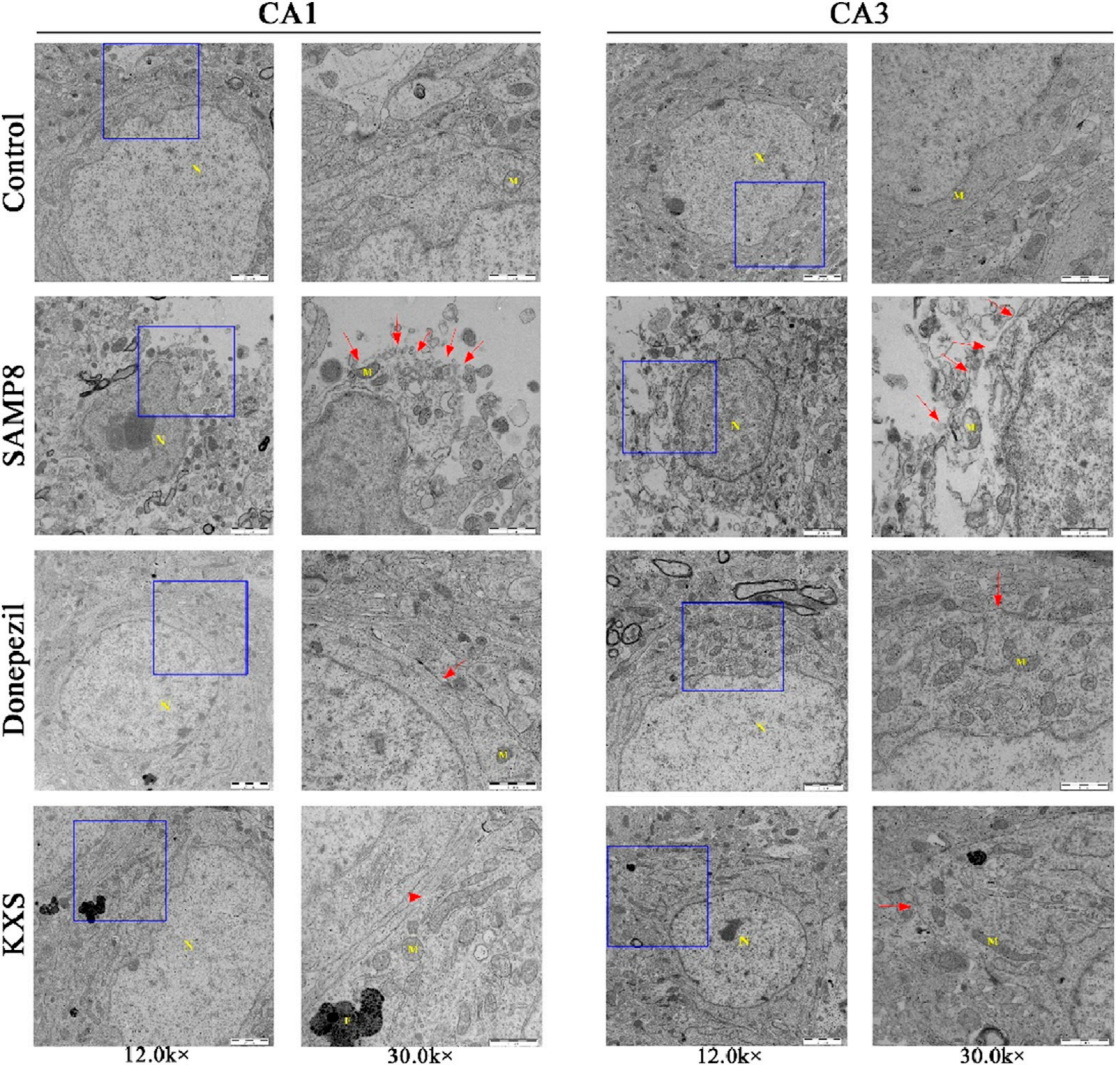
KXS attenuated the pyroptosis of hippocampal cells in SAMP8 mice (n = 3). Typical micrographs of TEM images of cells in the hippocampal CA1 and CA3 regions. N: nucleus, M: mitochondria, F: lysosomes. Magnification, 12.0 k ×, 30.0 k ×. Scale bars, 2 μm, 1 μm.

To examine cellular changes at the subcellular level, we employed TEM, which provides high-resolution imaging of cellular ultrastructure. Examination via TEM revealed pronounced ultrastructural changes in the hippocampal CA1 and CA3 regions of SAMP8 mice when compared to the control SAMR1 group, including edema expansion, multiple fracture breaks in the cell membrane, and most of the organelles in the cytoplasm were broken or disappeared. These are the specific features of pyroptosis, which is manifested as swelling and expansion of cells accompanied by the formation of plasma membrane pores, leading to cell rupture and the release of pro-inflammatory cytokines and cellular contents, exacerbating the inflammatory response. KXS and donepezil treatment substantially improved cellular ultrastructure, with only occasional cell membrane abnormalities observed (Figure 7). The results suggest that the pyroptosis of hippocampal cells in SAMP8 mice was significantly improved after KXS treatment.

### 3.8 KXS modulates inflammatory and pyroptotic pathways in SAMP8 mice

To further investigate the molecular mechanisms fundamental to the neuroprotective effects of KXS, we examined inflammatory markers and key components of the NLRP3/Caspase-1 pyroptosis pathway.

**FIGURE 8:**
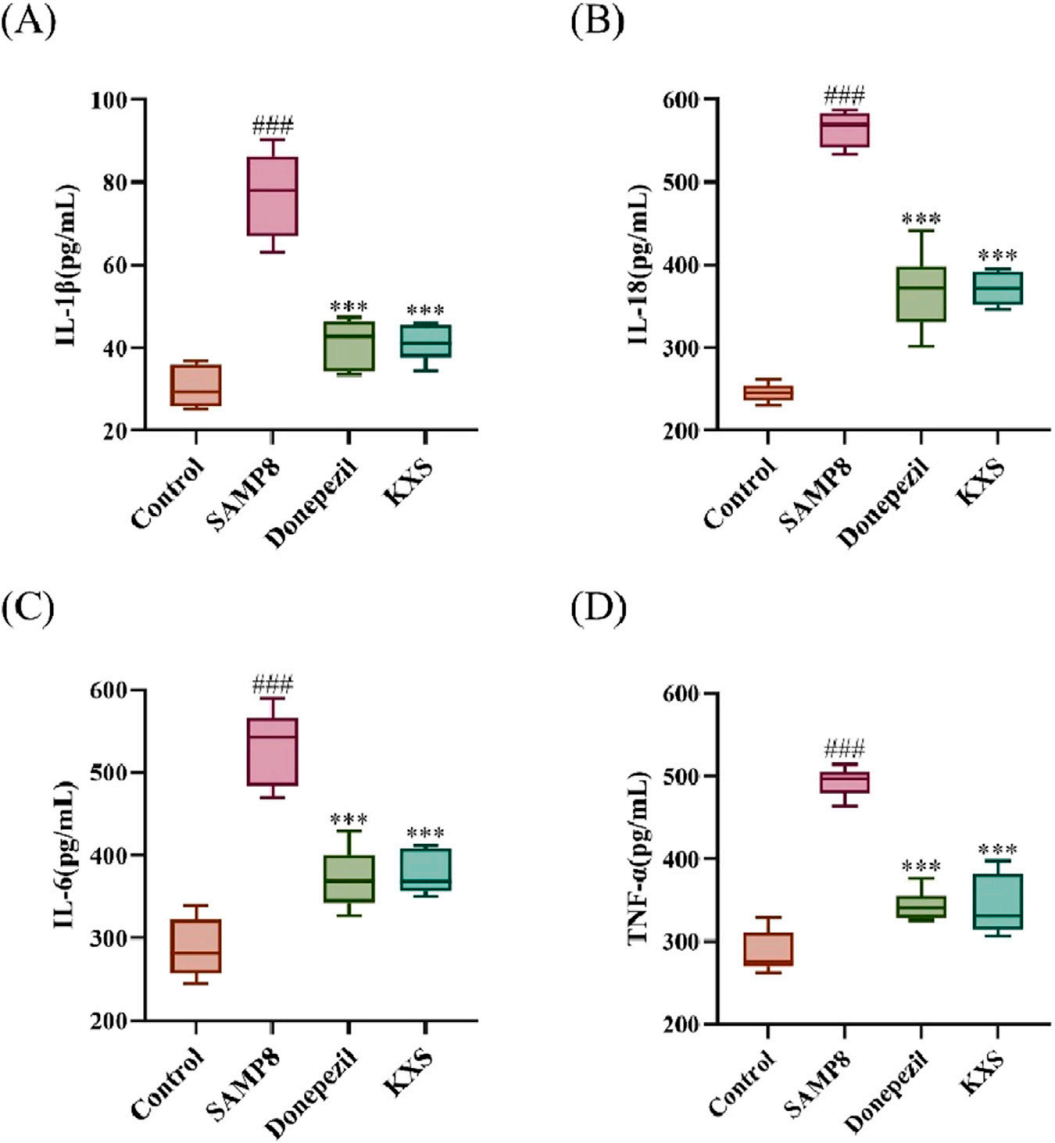
KXS reduced serum levels of inflammatory factors in SAMP8 mice (n = 10). **(A)** Concentration of IL-1β in serum. **(B)** Concentration of IL-18 in serum. **(C)** Concentration of IL-6 in serum. **(D)** Concentration of TNF-α in serum. Results are expressed as mean ± SD. ^###^ *P* < 0.001 vs. the control group; ***P < 0.001 vs. the SAMP8 group.

SAMP8 mice exhibited significantly elevated serum levels of pro-inflammatory cytokines (IL-1β, IL-18, IL-6, and TNF-α). After treatment with KXS and donepezil, serum levels of all four inflammatory factors in SAMP8 mice were markedly reduced (Figures 8A–D), demonstrating that KXS had a potent inhibitory effect on the inflammatory response of SAMP8 mice.

**FIGURE 9:**
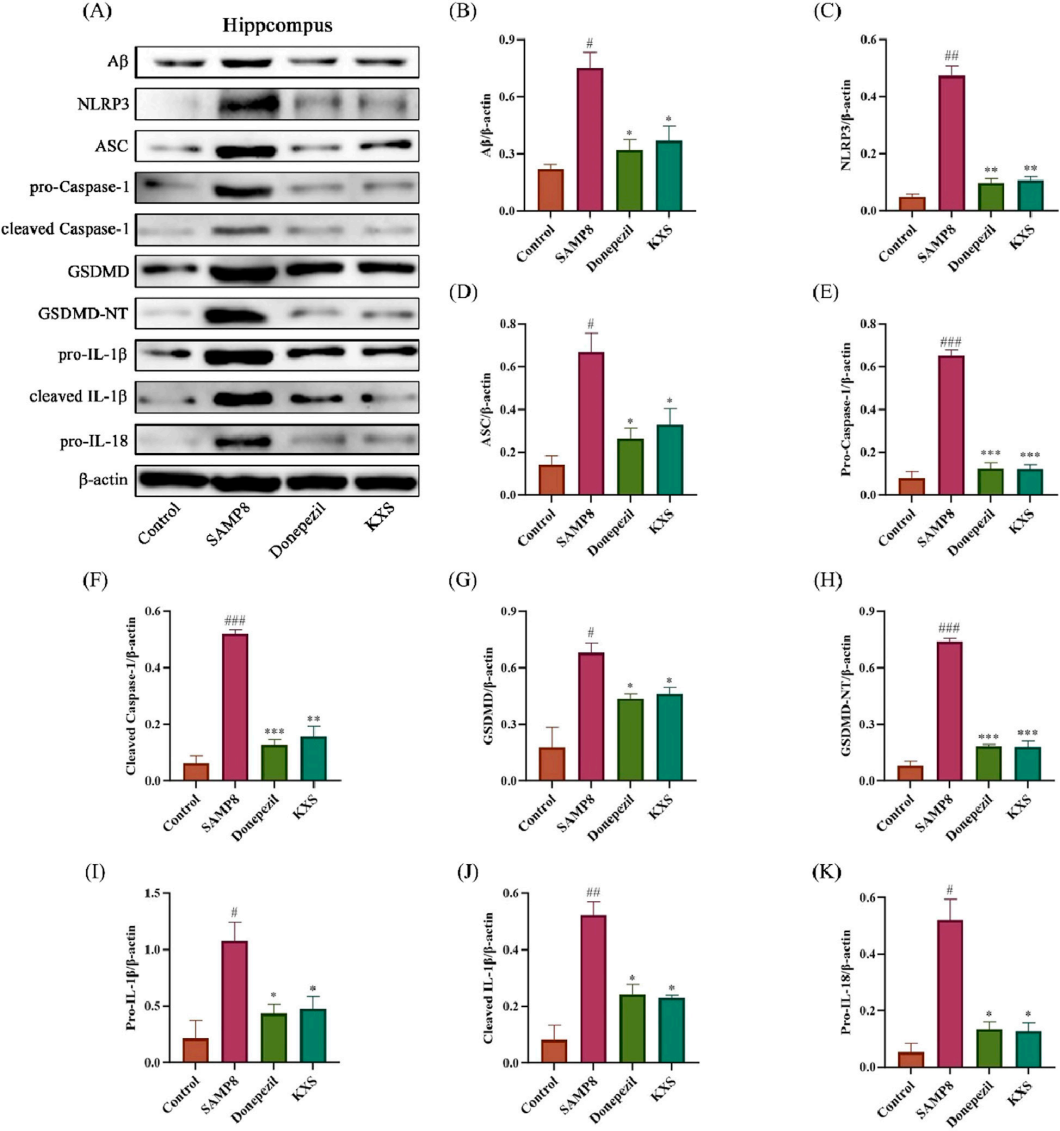
KXS modulated hippocampal Aβ protein and NLRP3/Caspase-1 signaling pathway-related protein expression in SAMP8 mice (n = 8). **(A)** Typical Western blot images showing protein expression of Aβ, NLRP3, ASC, pro-Caspase-1, cleaved Caspase-1, GSDMD, GSDMD-NT, pro-IL-1β, cleaved IL-1β, and pro-IL-18 from the hippocampus. **(B–K)** Quantification of relative protein levels. Results are expressed as mean ± SD. ^###^ *P* < 0.001, ^##^ *P* < 0.01, ^#^ *P* < 0.05 vs. the control group; ***P < 0.001, **P < 0.01, *P < 0.05 vs. the SAMP8 group.

Western blot analysis revealed significant upregulation of NLRP3 inflammasome components and downstream effectors in the classical pyroptosis NLRP3/Caspase-1 signaling pathway in the hippocampal regions of SAMP8 mice (Figures 9A–K). These changes included increased expression of Aβ, NLRP3, ASC, and pro-Caspase-1; enhanced activation of Caspase-1 (increased cleaved Caspase-1); elevated levels of pyroptosis executioner protein GSDMD and its active form GSDMD-NT; and increased production and processing of pro-inflammatory cytokines (IL-1β and IL-18). Remarkably, the expression levels of the relevant proteins were significantly reduced after treatment with KXS and donepezil, indicating a comprehensive inhibition of the NLRP3/Caspase-1 pyroptosis pathway (Figures 9A–K).

**FIGURE 10:**
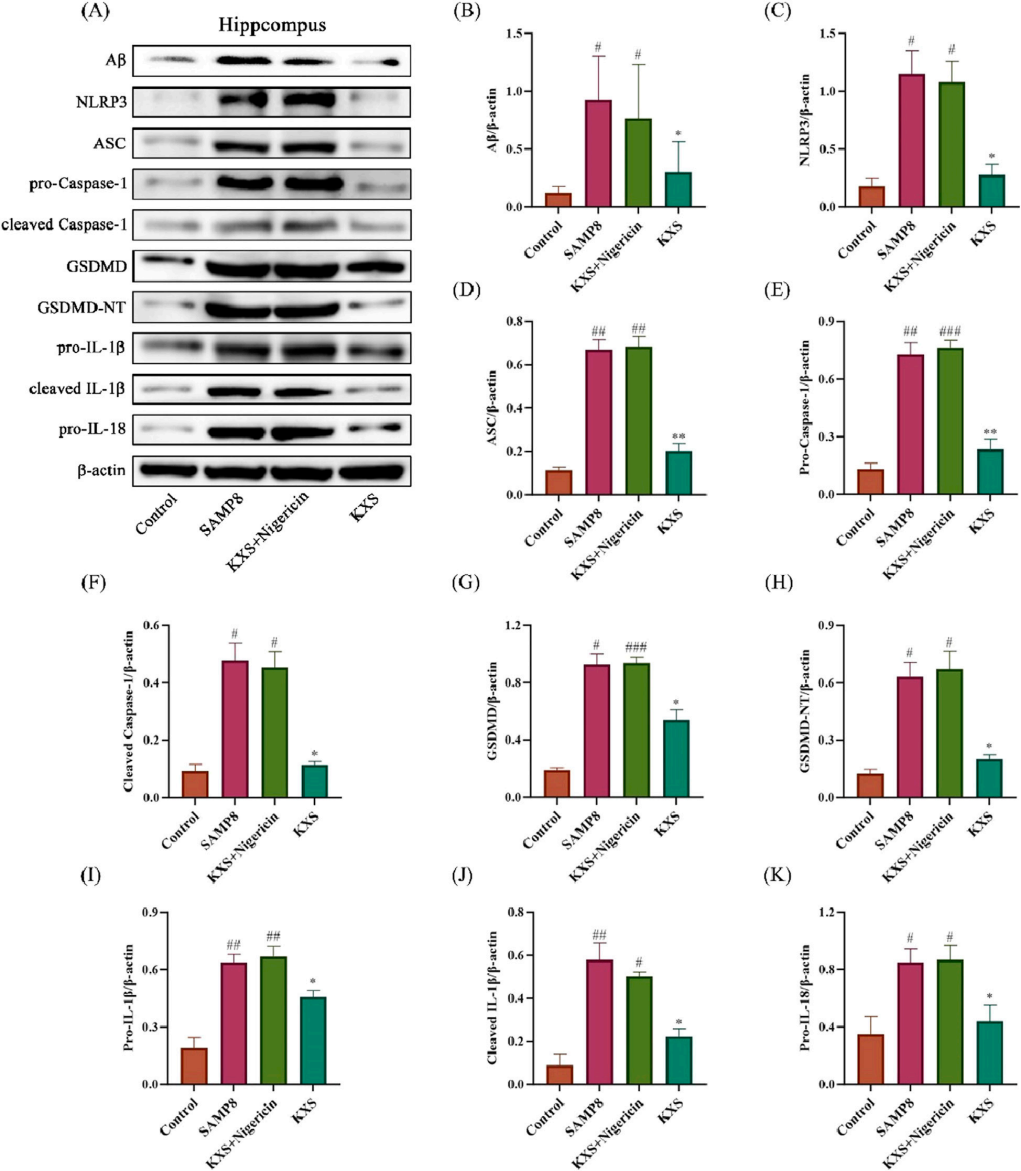
Effects of KXS on hippocampal Aβ protein and NLRP3/Caspase-1 signaling pathway-related protein expression in SAMP8 mice after Nigericin treatment (n = 8). **(A)** Typical Western blot images showing protein expression of Aβ, NLRP3, ASC, pro-Caspase-1, cleaved Caspase-1, GSDMD, GSDMD-NT, pro-IL-1β, cleaved IL-1β, and pro-IL-18 from the hippocampus. **(B–K)** Quantification of relative protein levels. Results are expressed as mean ± SD. ^###^ *P* < 0.001, ^##^ *P* < 0.01, ^#^ *P* < 0.05 vs. the control group; **P < 0.01, *P < 0.05 vs. the SAMP8 group.

To further elucidate the role of the NLRP3 inflammasome in KXS-mediated neuroprotection, we employed the NLRP3 activator Nigericin. Notably, Nigericin treatment abolished the suppressive effects of KXS on Aβ accumulation and NLRP3/Caspase-1 pathway activation (Figures 10A–K). These findings strongly suggest that KXS’s neuroprotective action is exerted, at least partially, through modulation of the NLRP3/Caspase-1 signaling pathway.

## 4 Discussion

In our research, we investigated the impact of Kai-Xin-San (KXS) on cognitive function, neuroinflammation, and pyroptosis using the SAMP8 mouse model, which is relevant to Mild Cognitive Impairment (MCI). Our findings demonstrate that KXS significantly improves cognitive performance, reduces Aβ deposition, attenuates neuroinflammation, and inhibits pyroptosis through modulation of the NLRP3/Caspase-1 signaling pathway. These results offer novel perspectives on the mechanisms underlying the therapeutic efficacy of KXS in MCI.

Donepezil is a second-generation cholinesterase inhibitor and is the only AD treatment-approved drug for marketing by both the FDA and the UK Medicines Agency. It is mostly used for the symptomatic treatment of MCI and mild to moderate AD. In recent years, a large number of studies have focused on the anti-inflammatory effects of donepezil (Yoshiyama et al., 2010; Haraguchi et al., 2017; Goschorska et al., 2018; Maroli et al., 2019). Therefore, donepezil may not only exert its cholinesterase inhibitory effect but also inhibit the neurodegenerative pathological changes of MCI through its anti-inflammatory effect. Therefore, in the present study, donepezil hydrochloride tablets were chosen as the experimental positive control drug to conduct an in-depth study of KXS on the role and mechanism of the inhibition of inflammatory response and hippocampal neuronal pyroptosis.

The behavioral tests revealed that KXS treatment notably enhanced spatial memory, working memory, and executive function in SAMP8 mice. These results align with earlier findings that establish the cognition-enhancing properties of KXS across diverse models of cognitive deficits (Luo et al., 2020; Wang et al., 2020). The multi-domain cognitive improvements observed in our study suggest that KXS may have broad neuroprotective effects, potentially targeting multiple aspects of MCI pathology.

First, KXS reduces Aβ deposition and modulates neuroinflammation. A key finding of our study is the significance of hippocampal Aβ deposition following KXS treatment. This aligns with the growing body of evidence implicating Aβ accumulation as a central pathogenic mechanism in MCI and AD (Hardy and Selkoe, 2002). The ability of KXS to reduce Aβ deposition may be attributed to its effects on Aβ production and clearance pathways, as suggested by previous studies (Sun et al., 2021).

Neuroinflammation is the inflammatory response in the CNS that is usually caused by various pathological injuries including infection, trauma, ischemia, and toxins. It is one of the important pathogenic mechanisms of MCI. In particular, chronic inflammation involving activated microglia increases the levels of pro-inflammatory factors that can penetrate the blood-brain barrier and aggravate brain inflammation. This would potentially amplify the amyloid cascade reaction, leading to Aβ deposition and neurotoxicity (Lee et al., 2021). In turn, excess deposited Aβ may accelerate the activation of microglia and exacerbate the inflammatory response. This vicious cycle of amyloid deposition and neuroinflammation leads to neuronal degeneration, which amplifies neuronal dysfunction and accelerates the pathological transformation of MCI to AD (Nordengen et al., 2019).

Here, we observed that KXS administration substantially lessened the deposition of hippocampal amyloid and decreased serum concentrations of pro-inflammatory cytokines (IL-1β, IL-18, IL-6, and TNF-α), and decreased microglial activation in the hippocampus. These findings suggest that KXS exerts potent anti-inflammatory effects, potentially breaking the vicious cycle between Aβ accumulation and chronic neuroinflammation that characterizes MCI progression. The ability of KXS to modulate both Aβ deposition and neuroinflammation simultaneously highlights its multi-target approach, which may be particularly advantageous in addressing the complex pathology of MCI.

Microglia, which are derived from yolk sac fetal macrophages, are major players in the neuroinflammatory response (Norris and Kipnis, 2019). By employing different regulatory networks, microglia exert important roles in the pyroptosis of nerve cells and immune monitoring (Ahmad et al., 2022; Morató et al., 2022). Activated microglia internalize pathogenic substances and degrade them through various intracellular pathways, which usually subside after the elimination of the immune stimulus (Subhramanyam et al., 2019). Microglial cells located in the MCI brain, however, are characterized by functional vulnerability and sustained activation, which may contribute to the initiation of MCI pathogenesis (Cai et al., 2022). In this research, we demonstrated that KXS can reduce the inflammatory activation of microglia, thereby attenuating the inflammatory response.

It has been shown that reactive microglia are tightly co-localized with amyloid plaques in the brains of MCI patients (Tan et al., 2020). Studies also found that pro-inflammatory cytokines produced by microglia can upregulate the level of β-secretase, suggesting microglia not only indirectly promote Aβ production, but also amyloid plaque formation (Leng and Edison, 2021). In addition, overactivation of NLRP3 inflammasome in microglia can exacerbate Tau protein hyperphosphorylation and neuro progenitor fiber tangles (Heneka, 2017; Li D.-D. et al., 2022). Our findings align with the above findings and show that KXS ameliorates the abnormal pathological changes in hippocampal cytopathic alterations and cell morphology in SAMP8 mice.

A novel finding of our study is the ability of KXS to inhibit pyroptosis in hippocampal neurons of SAMP8 mice. Pyroptosis is a distinct type of programmed cell death characterized by the emission of pro-inflammatory cytokines and subsequent inflammatory reactions (Kesavardhana et al., 2020). Activated microglia can trigger excessive NLRP3 inflammasome activation, which is underlying the activation of pyroptosis (Sbai et al., 2022). The NLRP3 inflammasome complex consists of three primary elements: the NLRP3 sensor, apoptosis-associated speck-like protein containing CARD (ASC), and effector protein Caspase-1. Once activated, Caspase-1 processes GSDMD, resulting in GSDMD-N-terminal (GSDMD-NT), which facilitates membrane pore formation for the secretion of mature IL-1β and IL-18, thereby triggering inflammation (Cai et al., 2021; Li M. et al., 2021). An increasing number of researchers have confirmed that the NLRP3 inflammasome significantly contributes to MCI pathogenesis. It has been shown that either recombinant Aβ_1-42_ or Tau proteins can activate NLRP3 inflammasome, trigger pyroptosis, and induce IL-1β release (Stancu et al., 2019; Han et al., 2020). The involvement of pyroptosis in MCI and AD pathogenesis is an emerging area of research.

Our findings suggest that KXS was able to significantly reduce the development of pyroptosis by significantly ameliorating cell expansion and distension. KXS may exert its neuroprotective effects at least in part by inhibiting the NLRP3/Caspase-1-mediated pyroptosis pathway. KXS treatment significantly inhibited the assembly and activation of inflammatory, reduced the levels of NLRP3 inflammasome key components (NLRP3, ASC, pro-Caspase-1) and its downstream effectors (cleaved Caspase-1, GSDMD, GSDMD-NT), and decreased the maturation of Caspase-1 and the secretion of IL-1β, IL-18. This inhibition of the pyroptotic pathway corresponded to a decrease in the generation of pro-inflammatory cytokines (IL-1β, IL-18) and improved cellular ultrastructure. Interestingly, the impacts of KXS on the NLRP3/Caspase-1 pathway were abolished by treatment with Nigericin, a potent NLRP3 activator. This observation further supports the specificity of KXS action on this pathway and suggests that NLRP3 inhibition may be a critical mechanism underlying its therapeutic effects. We further clarified that the anti-inflammatory effect of KXS was mainly achieved by inhibiting the NLRP3/Caspase-1 signaling pathway.

## 5 Conclusion

In conclusion, our research offers novel perspectives on the mechanisms underlying the therapeutic effects of KXS in MCI. We propose a comprehensive mechanism for KXS action in MCI that integrates its effects on the reduction of Aβ deposition, attenuation of neuroinflammatory injury, and modulation of NLRP3/Caspase-1 signaling pathway to attenuate hippocampal pyroptosis. These findings not only expand our understanding of KXS action but also underscore the potential of traditional Chinese medicine approaches in addressing complex neurodegenerative disorders. While our research delivers meaningful findings of the mechanisms through KXS action in MCI, it is worth to mention that the specific bioactive components of KXS responsible for its observed effects remain to be identified. Future studies should focus on isolating and characterizing these components to optimize therapeutic strategies.

## Data Availability

The raw data supporting the conclusions of this article will be made available by the authors, without undue reservation.

## Abbreviations

KXS, Kai-Xin-San; MCI, mild cognitive impairment; AD, Alzheimer’s disease; Aβ, β-amyloid; NLRP3, NOD-like receptor protein 3; SAMP8, senescence-accelerated prone 8; FJC, fluoro-Jade C; IHC, immunohistochemistry; TUNEL, terminal labeling; ELISA, enzyme-linked immunosorbent assay; GSDMD, gasdermin D; MWM, Morris water maze; NOR, novel object recognition; H&E, hematoxylin-eosin; TEM, transmission electron microscopy; Iba-1, ionized calcium-binding adaptor molecule-1; CNS, central nervous system; ASC, apoptosis-associated speck-like protein containing CARD; GSDMD-NT, GSDMD-N-terminal.
